# The Malaria-High Blood Pressure Hypothesis

**DOI:** 10.1161/CIRCRESAHA.116.308763

**Published:** 2016-06-23

**Authors:** Anthony O. Etyang, Liam Smeeth, J. Kennedy Cruickshank, J. Anthony G. Scott

**Affiliations:** From the Department of Epidemiology and Demography, KEMRI-Wellcome Trust Research Program, Kilifi, Kenya (A.O.E., J.A.G.S.); Department of Infectious Disease Epidemiology (A.O.E., J.A.G.S.), and Department of Non-Communicable Disease Epidemiology (L.S.), London School of Hygiene and Tropical Medicine, London, United Kingdom; and Cardiovascular Medicine Group, Division of Diabetes and Nutritional Sciences, King’s College, London, United Kingdom (J.K.C.).

**Keywords:** arterial stiffness, blood pressure, epidemiology, inflammation, malaria

## Abstract

**Rationale:**

Several studies have demonstrated links between infectious diseases and cardiovascular conditions. Malaria and hypertension are widespread in many low- and middle-income countries, but the possible link between them has not been considered.

**Objective:**

In this article, we outline the basis for a possible link between malaria and hypertension and discuss how the hypothesis could be confirmed or refuted.

**Methods and Results:**

We reviewed published literature on factors associated with hypertension and checked whether any of these were also associated with malaria. We then considered various study designs that could be used to test the hypothesis. Malaria causes low birth weight, malnutrition, and inflammation, all of which are associated with hypertension in high-income countries. The hypothetical link between malaria and hypertension can be tested through the use of ecological, cohort, or Mendelian randomization studies, each of which poses specific challenges.

**Conclusions:**

Confirmation of the existence of a causative link with malaria would be a paradigm shift in efforts to prevent and control hypertension and would stimulate wider research on the links between infectious and noncommunicable disease.

Age standardized mean blood pressures (BPs) are higher in many parts of Asia and sub-Saharan Africa than in high-income countries.^1^ Despite the high burden of cardiovascular disease in low- and middle-income countries (LMIC), few studies have examined their pathophysiology or treatment.^2,3^ Although demographic and lifestyle changes including urbanization^4^ contribute substantially to the burden of hypertension in LMICs, examining factors unique to or more prevalent in LMIC settings might reveal new pathophysiological mechanisms that could aid efforts to control the condition.

**Editorial, see p 7**

**In This Issue, see p 2**

The rise of cardiovascular disease in LMIC is occurring against the background of continuing high burden of infectious diseases.^5,6^ Several studies in more developed settings have reported links between infectious or inflammatory conditions and cardiovascular disease. In this article, we outline the hypothesis that hypertension, the leading risk factor for death in LMIC,^7^ could be linked to one of the leading infectious conditions in the same region, malaria.

## The Malaria–Hypertension Hypothesis

We postulate that malaria contributes to the burden of hypertension in LMIC in the following ways:

1. Malaria in pregnancy leads to low birth weight through pathophysiologically connected mechanisms.^8^ In areas with high malaria endemicity where women are likely to have acquired immunity to prevent most febrile episodes, low birth weight results from fetal growth restriction, which is a consequence of impaired uteroplacental blood flow^9^ and maternal anemia (which is itself because of malaria).^10,11^ Febrile malaria episodes that are more likely among women with low immunity are thought to induce uterine contractions, which are mediated by elevated levels of tumor necrosis factor-α leading to preterm birth.^12,13^ Malaria is also associated with hypertensive disorders of pregnancy such as gestational hypertension and preeclampsia in young primigravid women,^14–16^ and these are risk factors for low birth weight.^17^ Low birth weight children have an increased incidence of hypertension in later life.^18–21^ In a study conducted in Ibadan, Nigeria, infants of mothers who experienced malaria during pregnancy had a higher increase in BP levels during the first year of life compared with those who did not.^22^ Because BP levels track strongly through to adulthood, such differences could significantly influence the prevalence of adult hypertension.^23–25^ By virtue of its association with hypertensive disorders of pregnancy that are themselves risk factors for essential hypertension in women,^26,27^ malaria likely contributes to an intergenerational vicious cycle of disease susceptibility because hypertensive parents bear children who develop hypertension more frequently.^28,29^

2. Malaria is associated with stunting and malnutrition in childhood,^30,31^ which predisposes to the development of hypertension in later life.^19,23,32^

Although the biological pathways have not been fully characterized, postulated mechanisms involved in the development of hypertension after stunting and chronic malnutrition include reduced nephron numbers^18^ and premature senescence in the kidney, which is particularly prominent when there is rapid weight gain after growth restriction.^33^ In addition, Jamaican survivors of severe acute malnutrition in childhood were found at the age of 30 years to have markedly smaller left ventricular outflow tracts with reduced cardiac output in the presence of elevated peripheral resistance, a pattern of changes that is likely to lead to hypertension in later life.^34^

3. Malaria is a cause of chronic inflammation,^35^ and inflammation predisposes to cardiovascular diseases in high-income countries.^36^ In a prospective study of 20 525 female US health professionals, there was a linear relationship between baseline C-reactive protein levels and incident hypertension.^37^ Patients with inflammatory bowel disease and rheumatoid arthritis have increased arterial stiffness, which precedes hypertension.^38–40^ The link between inflammatory conditions and hypertension may be related to perturbations in the levels of endothelial-based growth factors. Angiopoietin-2 (Ang-2) is a multimeric ligand of the Tie 2 receptor, part of a vascular-specific tyrosine kinase signaling pathway that is essential for vessel development and stability.^41^ Ang-2 is predominantly secreted by endothelial cells and some smooth muscle cells in many inflammatory and angiogenic states (Figure). Ang-2 levels are elevated in children with severe malaria in several different settings^35,42–45^ and in returning travellers infected with malaria.^46^ Ang-2 levels predict cardiovascular disease in children with chronic kidney disease.^47^ Although no causal association has been established, several studies have demonstrated an association between Ang-2 levels and arterial stiffness and BP in adults.^48,49^

**Figure.**
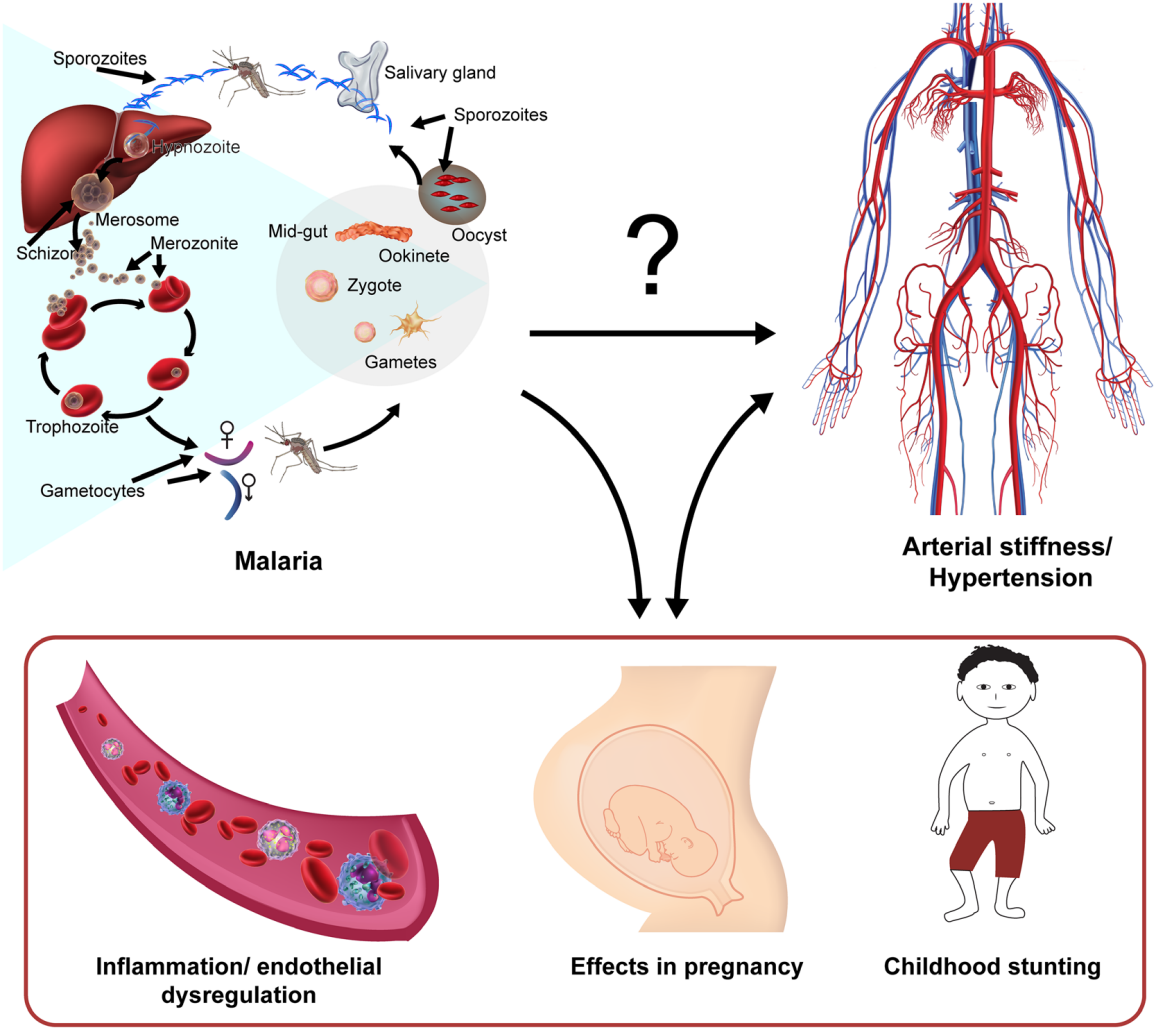
The malaria-high blood pressure hypothesis. Malaria is known to cause low birth weight, inflammation as well as stunting. All these factors have been separately associated with the development of high blood pressure in high-income countries. Studies are needed to confirm whether malaria contributes to the development of high blood pressure in low- and middle-income countries.

## Testing the Hypothesis

### Observational Studies

Ecological studies examining BP levels in relation to malaria incidence are hampered by the lack of finely scaled data on the relevant BP distributions. A worldwide study on BP levels had scarce raw data on BP from sub-Saharan Africa, where most malaria endemic countries are situated.^1^ In contrast, there are good epidemiological data on the spatial and temporal distribution of malaria.^50^

Although traditional case–control studies (with malaria as the exposure and hypertension as the outcome) could provide an efficient way to test the hypothesis, they are limited by the nonspecificity and nondurability of immunologic markers for malaria, a prerequisite for identifying individuals who have previously been exposed to malaria.^51–53^ This limitation also applies to the potential use of propensity scores^54^ to assemble groups that are comparable in their risk of malaria; in order to generate such scores reliable immunologic markers of malaria exposure would be needed.

A life-course epidemiological approach with longitudinal cohorts from the antenatal period or birth would allow the study of many of the postulated pathways through which malaria could be leading to hypertension in LMIC. Studies of pregnant women and children exposed to malaria in demographic surveillance systems with good quality ascertainment of exposure status are necessary. However, most demographic surveillance systems in LMIC were only set up in the last 15 years, and therefore may not have accumulated enough follow-up time to examine these outcomes, assuming that the biases of such retrospective studies can be overcome. Prospective surveillance, however, would also require long follow-up and be expensive to conduct.

### Randomized Intervention Studies

Because malaria is of such great public interest, there have been and will continue to be a succession of randomized controlled trials of interventions tested at population level, such as vaccines, bed nets, and drug treatments. For those interventions that turn out to be effective it might be possible to examine their effect on arterial stiffness and BP. Prospective studies of interventions known to be effective as well as studies of controlled human malaria infection^55^ cannot be used here because although they satisfy the criterion of having 2 randomized groups with and without malaria, the fact that the vascular outcomes being tested are potentially irreversible pose an ethical challenge.^56^ As with observational studies, extended follow-up might be needed as vascular differences in trial groups because of the effects of malaria may take longer to be apparent compared with the antimalarial effects of the interventions.

Animal studies are hampered by the fact that murine models of malaria and hypertension are imperfect approximations of their human analogs that have complex pathophysiology.^57,58^

### Genetic Studies

Mendelian randomization studies, where genetic polymorphisms are used as instrumental variables representing malaria exposure, would be particularly attractive for answering the question as they overcome many of the limitations of observational and intervention studies described above.^59^ Several hemoglobin polymorphisms provide some level of protection against malaria, including Hemoglobin C and S, and thalassemia.^60–62^ A comparison of arterial stiffness indices and BP in subjects with and without the polymorphisms in regions where they have been exposed to malaria in childhood would provide a robust test of the effect of malaria exposure on the development of hypertension.

An important prerequisite for using Mendelian randomization to make causal inferences about the effects of environmental exposures, is that the polymorphisms should not display pleiotropic effects, that is, they should not influence the outcome being studied through a pathway that is independent of the environmental exposure that they are being used as a proxy for.^59^ Some studies suggest that individuals with the sickle cell trait (SCT) are more likely to have cardiovascular events especially under extreme conditions, such as military training or athletics.^63,64^ A recent study among blacks found similar baseline BP in those with and without SCT, although on follow-up there was an increased incidence of chronic kidney disease in individuals with SCT.^65^ To exclude the possibility of confounding by pleiotropy, it may be necessary to include a control group that has not been exposed to malaria or use additional independent genetic polymorphisms, such as α thalassemia. If malaria causes hypertension and there are no pleiotropic effects of SCT, then one would expect to find higher BP in individuals without SCT compared with those with SCT in groups that have been exposed to malaria. Conversely, there would be no difference in BP based on trait status among those who have not been exposed to malaria.

## Implications of the Hypothesis

Current efforts at understanding hypertension in LMIC have had a narrow focus anchored on traditional risk factors identified among populations in high-income countries. Confirmation of the causative role of malaria in elevating BP would be of immense scientific interest and could lead to a paradigm shift on how to control hypertension in LMIC.

Malaria is only one of many infectious diseases that have a high incidence across LMICs. The inflammatory pathways activated in malaria infection are similar to those of other illnesses.^66^ It is therefore likely that if malaria contributes to the burden of hypertension through inflammation, the same could be true of other chronic infections, such as HIV and tuberculosis, providing a novel impetus for the study and control of these infections. Currently, most treatment for infectious illnesses is focused on eliminating the pathogen with little regard for modulating the inflammatory responses that might result in adverse vascular consequences later. Elucidating these inflammatory pathways and their consequences would pave the way for trials of adjunctive therapy such as statins or specific cytokine antagonists to prevent adverse vascular remodeling as a result of infection.

## Sources of Funding

A.O. Etyang, L. Smeeth, and J.A.G. Scott are funded by the Wellcome Trust (Fellowship numbers: 103951/Z/14/Z, 098532 and 098504). The Funders played no role in the preparation of this article.

## Disclosures

None.

## Novelty and Significance

### What Is Known?

- Malaria and high blood pressure are widespread in low- and middle-income countries.
- Malaria is known to cause low birth weight, stunting, and inflammation.
- Low birth weight, stunting, and inflammation are associated with the development of arterial stiffness and high blood pressure in developed countries.
